# Dynamics of Crowded Vesicles: Local and Global Responses to Membrane Composition

**DOI:** 10.1371/journal.pone.0156963

**Published:** 2016-06-16

**Authors:** Daniel A. Holdbrook, Roland G. Huber, Thomas J. Piggot, Peter J. Bond, Syma Khalid

**Affiliations:** 1 Bioinformatics Institute (A*STAR), 30 Biopolis Str, #07–01 Matrix, Singapore 138671, Singapore; 2 The Defence Science and Technology Laboratory, Porton Down, Salisbury, SP4 0JQ, United Kingdom; 3 Department of Biological Sciences, National University of Singapore, 14 Science Drive 4, Singapore 117543, Singapore; 4 School of Chemistry, University of Southampton, Southampton, SO17 1BJ, United Kingdom; London, UNITED KINGDOM

## Abstract

The bacterial cell envelope is composed of a mixture of different lipids and proteins, making it an inherently complex organelle. The interactions between integral membrane proteins and lipids are crucial for their respective spatial localization within bacterial cells. We have employed microsecond timescale coarse-grained molecular dynamics simulations of vesicles of varying sizes and with a range of protein and lipid compositions, and used novel approaches to measure both local and global system dynamics, the latter based on spherical harmonics analysis. Our results suggest that both hydrophobic mismatch, enhanced by embedded membrane proteins, and curvature based sorting, due to different modes of undulation, may drive assembly in vesicular systems. Interestingly, the modes of undulation of the vesicles were found to be altered by the specific protein and lipid composition of the vesicle. Strikingly, lipid dynamics were shown to be coupled to proteins up to 6 nm from their surface, a substantially larger distance than has previously been observed, resulting in multi-layered annular rings enriched with particular types of phospholipid. Such large protein-lipid complexes may provide a mechanism for long-range communication. Given the complexity of bacterial membranes, our results suggest that subtle changes in lipid composition may have major implications for lipid and protein sorting under a curvature-based membrane-sorting model.

## Introduction

The dynamic behavior of lipids and their interplay with membrane proteins is central to functional organization and compartmentalization within cells and organelles. Furthermore, lipid sorting and aggregation of integral membrane proteins can result in a crowded environment, which likely plays a role in structural, mechanical and functional aspects of such membranes. However, the processes that trigger the spontaneous sorting of these components, and the dependency upon properties such as membrane composition and curvature, are still poorly understood. The combined effects of the overall curvature and local lipid/protein hydrophobic mismatch in cell membranes have been suggested to be key drivers of protein aggregation [1, 2]and induced lipid sorting[3]. Concerted motions, where lipids move in loosely defined groups [4] and in complex with proteins [5, 6] are also likely to be important drivers of membrane structure and dynamics. Moreover, long wavelength undulations in membranes can have a measureable impact on local bilayer properties [7–9] and lead to selective sorting of lipids [10] which may thus modulate protein aggregation[11].

Molecular dynamics (MD) simulations have been used extensively as a tool for studying the behavior of membranes and their embedded proteins[12]. The computational cost of fully atomistic simulations typically reduces their usefulness in studying systems in which large-scale lipid sorting, protein clustering, or membrane curvature play a role. In this regard, coarse grained MD (CG-MD) simulations, which use a reduced representation of the molecular components, have been used to access events over longer, biologically-relevant time and length-scales, typically with pseudo-atomic resolution [12–15]. Indeed, CG-MD has been used, for example, to model vesicle formation [16, 17] and fusion[18, 19], transmembrane protein aggregation[2, 20], and to study the dynamics of viral envelope proteins[21].

**Fig 1:**
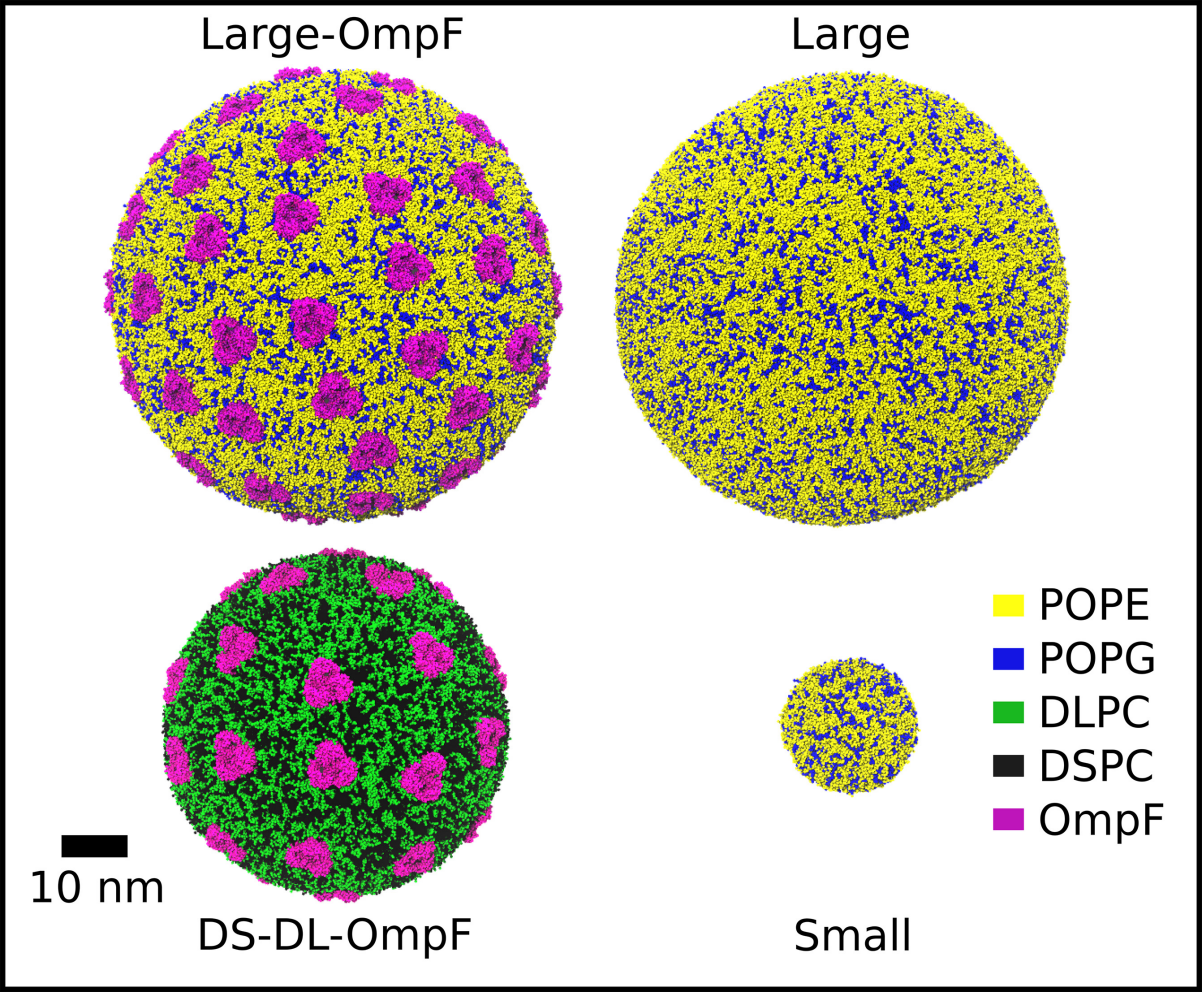
Vesicle systems and their global dynamics. The starting structures of the vesicle systems simulated in the study, as labeled, shown in spacefill representation with colors of different molecular species indicated inset.

Currently, there is limited information on the role of biologically relevant bilayer composition and curvature upon lipid sorting, and the spatial arrangement of embedded proteins within the crowded membrane environment. Moreover, the mechanisms by which associated static, structural and dynamic lipid/protein and protein/protein interactions are communicated within the membrane milieu are ill-understood, and have so far proven difficult to characterize even in simplified membrane models. To tackle this, the present study employs a CG simulation approach to study the effects of system size, protein content, and lipid composition upon protein-lipid interactions and bilayer undulations in unilamellar vesicles (Fig 1). These systems vary in their degree of complexity, ranging from a “simple” mixture of short- and long-tailed phoshpatidylcholine lipids to realistic models of the bacterial inner membrane, with vesicle diameters reaching those commonly studied in biology[22], i.e. > 50 nm. Within some of these vesicles, high concentrations of the trimeric bacterial outer membrane protein OmpF, a highly stable porin from *Escherichia coli*, were embedded to simulate the crowded biological membrane environment. The global and local properties of these vesicles over multi-microsecond timescales were subsequently investigated. A tendency for substantial selection and depletion of certain annular lipids around OmpF clusters was observed, whilst a spherical harmonic decomposition analysis revealed membrane undulations and overall vesicle dynamics dependent upon lipid composition, which may be important in determining protein aggregation. The protein-lipid motion was found to be correlated over substantially longer distances in our spherical systems than has been previously suggested for flat lipid bilayers.

## Results

### Simulation systems

The present study employs the MARTINI CG-MD force field [13] with modified CG protein parameters[23–25]. In total, the dynamics of four coarse-grained vesicle systems spanning three sizes and different lipid and protein compositions were investigated (Table 1, Fig 1). The “Small” vesicle had a diameter of ~16 nm, similar to that of previously reported simulations [19] whilst the “Large” systems represent biologically relevant scales of ~65 nm diameter [22]. A vesicle of intermediate radius (~25 nm) in the DS-DL-OmpF system contained a mixture of long-tailed, DSPC (1,2-distearoyl phosphatidyl choline), and short-tailed, DLPC (1,2-dilauroyl phosphatidyl choline) lipids in a 1:1 ratio. The Small, Large and Large-OmpF systems contained symmetric membranes composed of POPE and POPG lipids in a 3:1 ratio, representing a Gram-negative inner membrane environment [6, 26, 27]. Both the Large-OmpF and DS-DL-OmpF systems contained high concentrations of OmpF, with a ratio of ~481 and ~595 lipids per trimer, respectively. Other OMPs from *E*. *coli* have been shown to slow lipid diffusion up to 4 nm from the surface of proteins in CG-MD, but with the greatest impact occurring within 2 nm of the protein [6].

**Table 1:** The composition and properties of the vesicle systems.

System Name	Time (μs)	Average Radius (nm) ± SD	Total No. of Lipid and Solvent Molecules	Total No. of OmpF trimers	Mean Nearest Neighbour Distance* (xyz) ± SD (Å)	Mean Nearest Neighbour Distance* (great circle distance) ± SD (Å)
Small	9.2	8.4 ± 0.004	2,578 (3:1) POPE:POPG, 57,503 Water beads	0	5.97 ± 0.037	6.60 ± 0.055
Large	3.0	32.8 ± 0.003	41,248 (3:1) POPE:POPG, 1,761,721 Water beads	0	5.96 ± 0.040	6.25 ± 0.068
Large-OmpF	4.3	32.4 ± 0.13	30,788 (3:1) POPE:POPG, 1,917,163 Water beads	64	6.04 ± 0.039	6.45 ± 0.055
DS-DL-OmpF	6.4	24.3 ± 0.09	19,029 (1:1) DSPC:DLPC, 841,368 Water beads	32	6.40 ± 0.015	6.31 ± 0.057

* Mean nearest neighbor distance is calculated between lipids

### Protein aggregation

**Fig 2:**
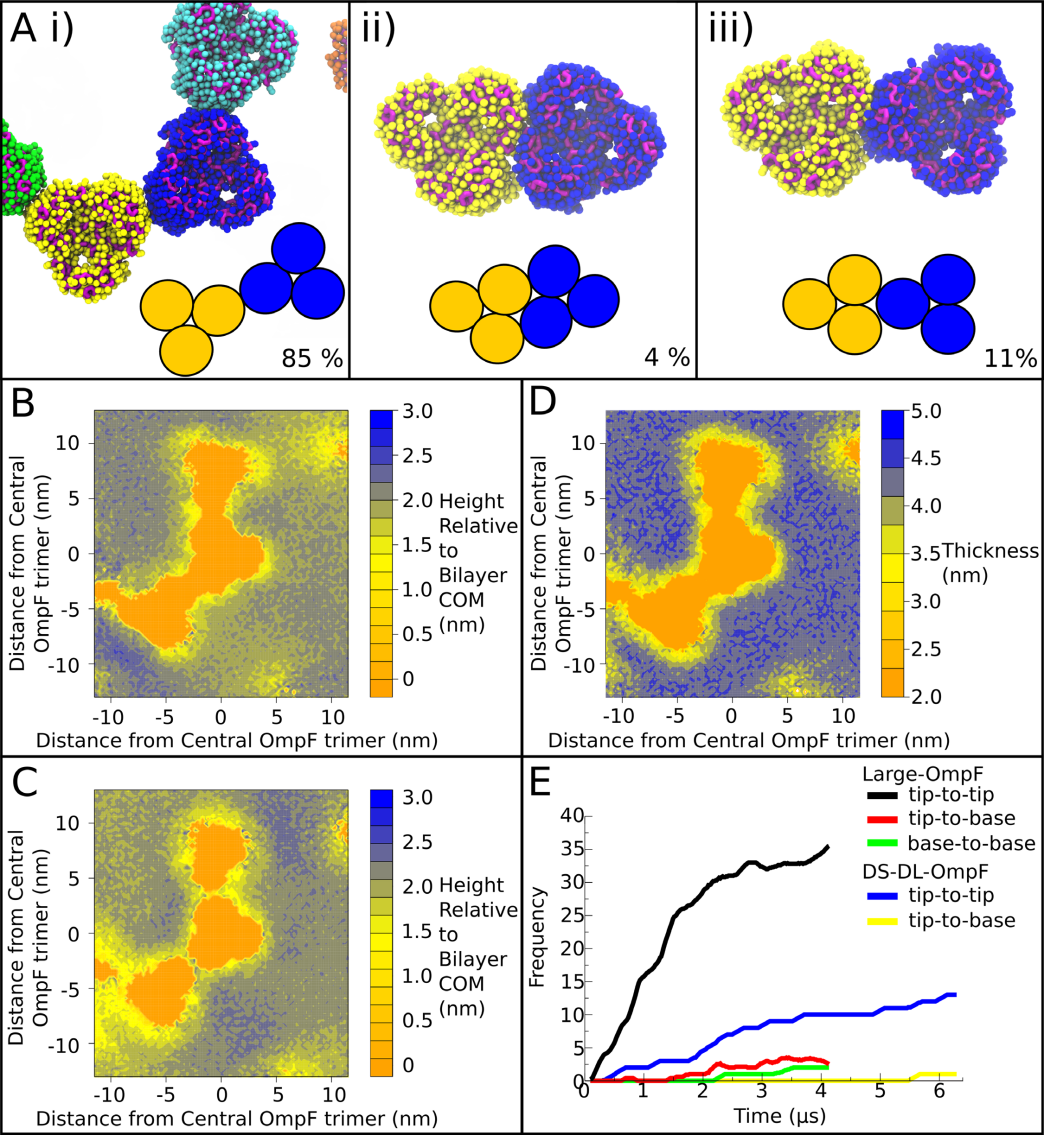
Protein aggregation and associated membrane thinning. **(A)** The different configurations of OmpF association observed within Large-OmpF and DS-DL-OmpF, namely: i) tip-to-tip, ii) base-to-base, and iii) tip-to-base, corresponding to the low-energy arrangements observed in [28]. The OmpF trimers are shown in spacefill representation with the backbone and sidechain particles indicated in different colors, and schematic representations of each low-energy arrangement included inset. The percent occurrence across all trajectories is shown for each configuration. **(B)** Contour map representing the height of the inner leaflet of the lipid bilayer, based on the phosphate particle positions, around a cluster of OmpFs in Large-OmpF. The bilayer COM is the position of the weighted average radius of the vesicle. The same cluster of OmpFs is shown in panel (A, i). **(C)** Contour map representing the height of the outer leaflet of the lipid bilayer for same cluster of OmpFs in (B). **(D)** Contour map representing the total thickness of the bilayer for the cluster of OmpFs in (B) and (C), calculated as the distance between the phosphates of the inner and outer leaflets of the bilayer. Distances in the x and y dimensions in (B), (C) and (D) are the summed great circle distances from point to point along the edge of the grid used to group phosphate particles. Due to the 2D projection of the phosphate positions, some distortion of the area is expected towards the corners. However, the distortion is limited, as the central portion, representing only half the surface of the cube face projection, has been shown in the figure. **(E)** Time-dependent aggregation of OmpF. The number of occurrences of the three types of trimer-trimer interface for OmpF in Large-OmpF and DS-DL-OmpF, presented as a function of time. A cutoff distance of 6 nm between the center of mass of each OmpF subunit with every other OmpF subunit was used to determine the number of interacting subunits for each trimer.

Starting from systems in which the proteins were placed at equidistant positions within the vesicle membrane, and in uncorrelated orientations with respect to one another, the OmpF trimers were observed to aggregate over the length of the trajectories. Previous studies have suggested that the OmpF trimer aggregates in three distinct fashions, termed “tip-to-tip”, “tip-to-base” and “base-to-base”, using both atomic force microscopy and molecular dynamics simulations [28]. All three of these forms of aggregation were observed in the Large-OmpF and DS-DL-OmpF simulations (Fig 2), in the ratios 44:5:2, for tip-to-tip, tip-to-base and base-to-base, respectively (Fig 2A). In the individual simulations, the ratios were 36:4:2 in Large-OmpF and 13:1:0 in DS-DL-OmpF. The aggregation proceeded rapidly, with 85% of the total observed interactions occurring within the first 3 μs of the trajectories (Fig 2E). A fully stable configuration of interactions was not achieved at the end of either the Large-OmpF or DS-DL-OmpF simulations, and aggregation continued until the final stages of the trajectories. Spontaneous inter-conversion between the three different types of association was not observed in either Large-OmpF or DS-DL-OmpF. Several chains of interacting OmpF trimers were formed, with the longest occurring in Large-OmpF and involving 8 trimers. The longest chain in DS-DL-OmpF involved 5 trimers. The preponderance of tip-to-tip interactions is consistent with the proposed mechanism of initial stages of OmpF association, whereby the tip-to-tip arrangement represents a loose initial interaction, and tip-to-base and base-to-base are the subsequent tighter associations. As a result of the curvature of the vesicle membranes, the tip-to-tip interactions between OmpF trimers involve only a small number of residues at the base of transmembrane β-strands that were exposed to in the inner leaflet of the bilayer. In the outer leaflet exposed surfaces, there is a gap of ~0.6 to 0.8 nm between the OmpF trimers, which is large enough for lipids to mediate interactions between the subunits. The tip-to-tip interactions buried on average 0.50 nm^2^ of solvent excluded volume from each protein, calculated using a probe radius of 0.26 nm [1]. This relatively small buried surface area, compared to typical protein-protein interfaces that bury >50.0 nm^2^ [29], may indicate that factors other than direct interaction between the trimers are involved in driving OmpF aggregation.

### Protein aggregates cause local membrane thinning and lipid sorting

**Fig 3:**
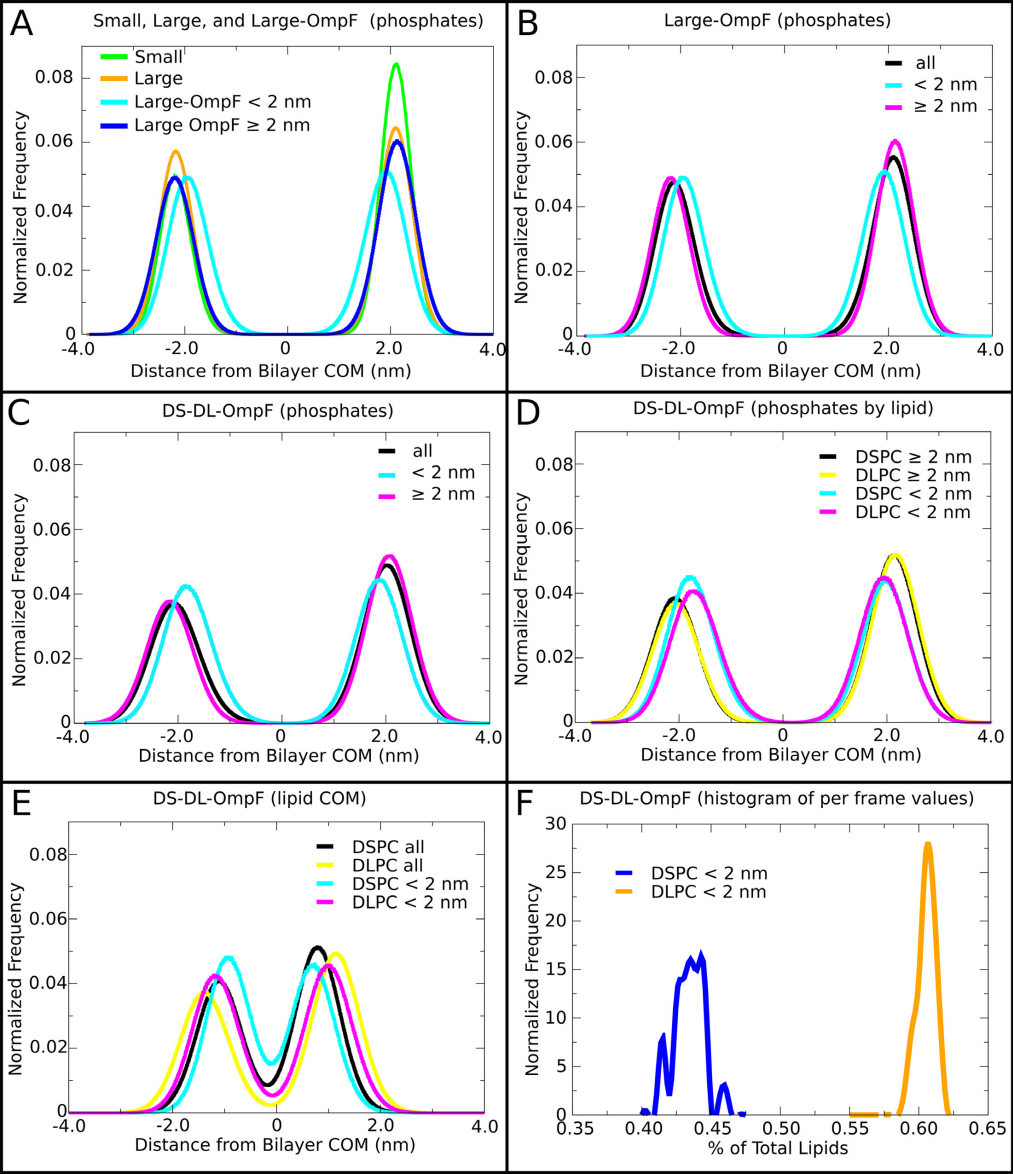
Vesicle membrane structure. The lipid phosphate particle positions are shown relative to the weighted radial center of the bilayer in Small, Large, DS-DL-OmpF and Large-OmpF. In (B), (C), (D) and (E) the lipids are separated based on whether they are close to OmpF (< 2nm), part of the bulk lipids (≥ 2nm), or considered altogether (all). In (C) and (D), the phosphate positions are shown for each lipid type in DS-DL-OmpF. In (E), the COM of the lipids is shown for each lipid type in DS-DL-OmpF. In (F), the per frame (spaced every 20 ns) percentage of lipids close to OmpF (< 2 nm) is shown separately for DLPC and DSPC. All the distributions, (A) to (F), have been calculated from frames originating from the second half of the trajectories.

To assess the origin of the tendency for protein aggregation, we analyzed the differences in membrane width associated with “local” annular and “non-local” bulk lipid layers, defined here as < 2 nm or ≥ 2 nm great circle distance from the protein surface, respectively. The interplay between OmpF and the water-bilayer interface was first examined, by measuring the positions of the lipid phosphate particles relative to the weighted COM of the bilayer. The presence of protein in the Large-OmpF system broadened the radial distribution of phosphate particles relative to the COM of the vesicle bilayer, with a standard deviation (SD) of 0.33 nm for Large and 0.40 nm for Large-OmpF. The average radial distance of the phosphates from the weighted center of the bilayer was smaller in Large-OmpF at 2.09 nm than Large at 2.13 nm. The influence of OmpF on the radial phosphate distribution was largely the result of local effects on the annular lipids surrounding OmpF, where the bilayer thins relative to the bulk lipids, by between 0.3 to 0.7 nm, particularly at the “tips”, and to a lesser extent at the interface of the trimer (Figs 2B, 2C, 2D and 3). In addition, a small degree of thinning of between 0.2 and 0.5 nm nm extended 2 to 5 nm from the protein surface into bulk, at the interface between clusters of OmpF trimers (Fig 2D). However, on a global scale, the influence of OmpF on annular lipid membrane width appeared not to extend much beyond the local environment around OmpF. Indeed, the removal of the annular lipids within a 2 nm great circle distance of the OmpF trimer yielded radial phosphate distributions in each leaflet of Large-OmpF whose SDs were 0.37 nm, closer to the values for the Large simulation. The distance between the inner and outer leaflet peaks of the bulk lipids in Large-OmpF was 4.3 nm (Fig 3B), which is comparable to the Large system also at 4.3 nm (Fig 3A). The distance between the peaks of annular lipids by comparison was 3.8 nm. The influence of OmpF on local lipid structure is also supported by the radial phosphate distribution of the Small system, which, despite a significant reduction in size, yielded a SD of 0.31 nm, and distance between peaks of 4.2 nm, similar to that of the Large system.

**Fig 4:**
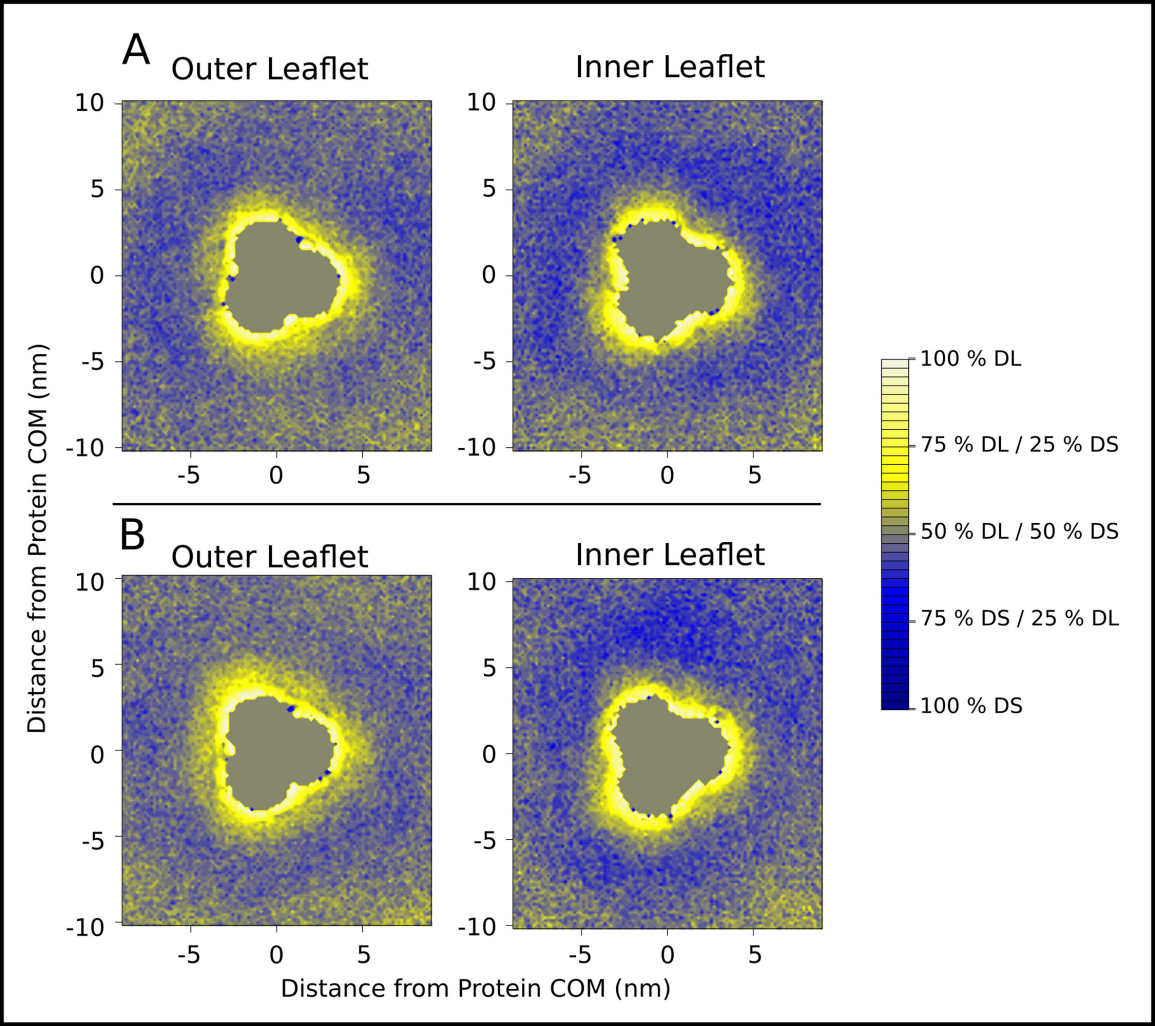
Lipid sorting around protein. Contour map indicating the relative occurrence of DLPC or DSPC lipids around an OmpF trimer in DS-DL-OmpF. The data are averaged over the final 200 ns of the trajectory. The distribution of lipids has been calculated separately for two isolated OmpF trimers, shown in (A) and (B), from the DS-DL-OmpF vesicle. Neither of the OmpF trimers interacted with any other protein during the simulations. The scale shows the relative frequency of finding either DSPC or DLPC at different positions around the OmpF trimer. The outer and inner leaflets are shown separately. Distances in the x and y dimensions are the summed great circle distances from point to point along the edge of the grid used to group phosphate particles. Due to the 2D projection of the phosphate positions, some distortion of the area is expected towards the corners. However, the distortion is limited, as the central portion, representing only half the surface of the cube face projection, has been shown in the figure.

The influence of lipid tail composition upon the distribution of annular lipids was also investigated. Thus, the DS-DL-OmpF system, containing an equal mixture of DS and DL phospholipids, exhibited a similar thinning of the bilayer around OmpF (Fig 3C), with a distance between the peaks in phosphate distributions of 4.2 nm for bulk lipids and 3.7 nm for annular lipids. Due to the high density of protein in the vesicle, approximately 27% of the lipids were found to be annular lipids across the whole the trajectory. Of these, 16% were DLPC and 11% DSPC, indicating a clear preference for OmpF to interact with shorter tailed lipids. An analysis of the relative probability of finding DLPC or DSPC at different positions around the protein (Fig 4) correlated with the thinning of the bilayer observed in Large-OmpF. Indeed, a preference for the shorter tailed DLPC lipids was observed at small distances, <2 nm, from the protein surface (Fig 3D and 3F). At even closer distances, <0.5 nm, from the protein surface there was increased selection for DLPC lipids, with greater than 75% of lipids found to be DLPC. The lipid-exposed surface of the OmpF trimer (Fig 4) was in some instances punctuated by sites of high levels of DSPC occurrence (100% DSPC). Many of these sites appeared to be present at the tips on the OmpF trimers, and in general corresponded to histogram bins that remained unoccupied by lipids for much of the simulation. In two cases, there was an apparent preference for DSPC lipids near the interface of the subunits of the trimer (Fig 4). This would correlate with less thinning of the bilayer at these positions in Large-OmpF, and with reports of a thickening at this position in other vesicle systems [30]. At greater distances from the OmpF surface, between 2 nm and 6 nm, there was a ring of slight enrichment of DSPC lipids, which largely dissipated beyond 6 nm from the protein.

### Membrane lipids are densely packed in the OmpF annular layer

The coupling of annular lipids may facilitate long-range communication between OmpF trimers, allowing them to associate in particular patterns of arrangement. Therefore, the properties of annular lipids in close proximity to OmpF were characterized in both protein-containing vesicles, by calculating the mean nearest neighbor distance (MNND) in three-dimensional space between pairs of lipids. The properties of lipids in the non-protein containing vesicles, Small and Large, were also compared to the bulk lipid in Large-OmpF and DS-DL-OmpF. Due to the extensive averaging over space and time, the SDs from the average values were small, < 0.06 Å (Table 1). The size of the system had little influence on the 3D spatial organization of lipids. Thus, there was less than a 0.001 nm difference in MNND between the Small (~0.597 nm) and Large (~0.596 nm) pure-lipid systems (Table 1). In contrast, the presence of 64 OmpF trimers in the Large-OmpF system appeared to have a much larger impact on the MNND, where the value averaged over the entire trajectory increased by 0.008 nm over the Large system. This measure may have been affected by a decrease in vesicle size in the OmpF containing simulations (Table 1, S1A Fig), with MNND values decreasing by 0.002 nm in Large-OmpF (S1B Fig) between the first and second halves of the trajectory, before stabilizing over the remainder of the simulation. Nevertheless, a large proportion of the increased MNND can be attributed to the more loosely packed lipids, primarily those in the bulk phase. Thus, the removal from the calculation of the annular layer around protein yields a MNND of 0.612 nm for the full trajectory and 0.609 nm for the second half of the trajectory, which is substantially larger than the MNND for all lipids of Large-OmpF (Table 1). This reveals that the annular lipids were packed more closely together than the bulk lipids, despite the lower overall lipid density.

The Mean Nearest Neighbour Great Circle distance (MNNGC) was also investigated (Table 1, S1C Fig), calculated as the great arc that connects the COM of each lipid. This measure is analogous to the surface (2D) MNND that is often calculated for planar bilayer systems, though it is sensitive to size of the vesicle, as is evident in the Small system whose MNNGC (0.660 nm) is greater than Large (0.625 nm), despite their similar MNNDs. Similarly to the trends in MNND, a lengthening of the MNNGC was observed for bulk lipids in Large-OmpF (0.671 nm) in comparison with all lipids together (0.645 nm), confirming the tighter packing of annular lipids. The MNNGC of the Large system was 0.02 nm smaller than in Large-OmpF, at 0.625 nm, indicating that the bulk lipids of the latter system were lower in density.

### Motion of densely packed annular layer lipid and protein are correlated

The DS-DL-OmpF system also displayed a larger MNND, with a ~0.04 nm increase over all other systems. This may be attributed to a mismatch in length of two lipids, leading to each having different COM positions relative to the COM of the bilayer (analogous to the height within a planar bilayer), hence leading to different average radial positions within the vesicle. Indeed, the COM of DSPC lipids was on average 0.3 nm closer to the center of the bilayer than DLPC lipids (Fig 3E), whereas the COMs of the POPE and POPG lipids in the Small, Large and Large-OmpF systems exhibited similar distances to one another from the center of the bilayer. Similarly to Large-OmpF, the bulk lipids of DS-DL-OmpF had a larger MNND (0.647 nm) and MNNGC (0.659 nm) than for all the lipids together, where the MNND was 0.640 nm and MNNGC was 0.631 nm, meaning that the annular lipids that are composed mostly of DLPC, as described above, affected the measure of compactness of the bilayer. The shortening of these distances for annular lipids results from the COMs of DL lipids occupying similar surfaces from the center of the vesicle (Fig 3E).

**Fig 5:**
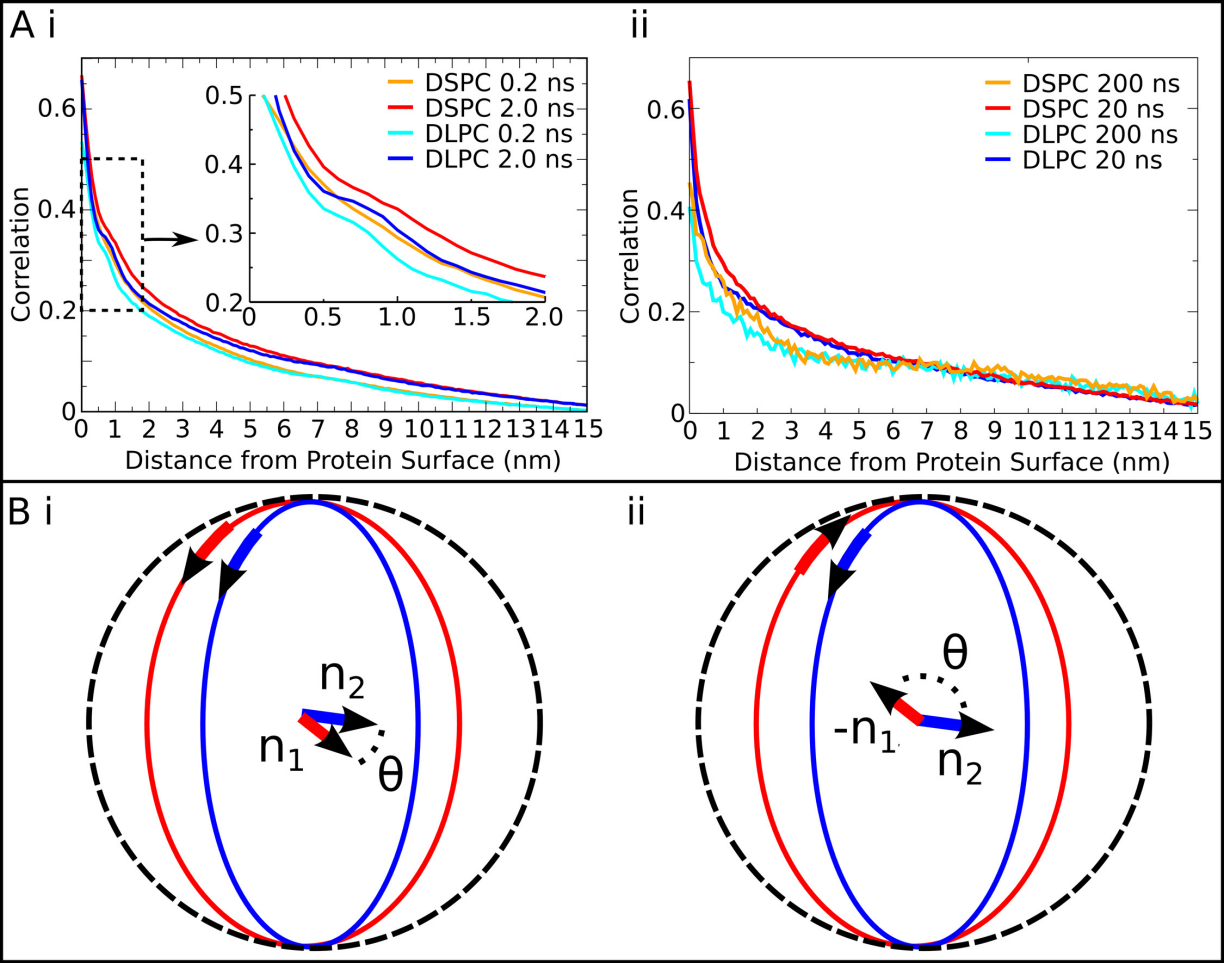
Protein aggregate associated lipid sorting. **(A)** i) The direction correlation of lipid and protein displacements as a function of distance from the proteins after 0.2 ns or 2.0 ns time jumps. The data have been averaged for all proteins and over the full length of the trajectories. The correlations are shown separately for DLPC and DSPC lipids; ii) The direction correlation of phospholipids (DLPC and DSPC) and OmpF displacements for time jumps of 20 ns or 200 ns are shown as a function of the phospholipid distance from the surface of the protein. The correlation was calculated as an average over all lipids and protein movements over the course of the trajectories. Fewer data points exist for longer time jumps, leading to incomplete convergence of the correlation measure. **(B)** Schematic description of the calculation of the direction correlation. The direction correlation is related to the angle between the axes of rotation of two lipids, red (n_1_) and blue (n_2_), traveling on great circles, (i). In the opposite direction of travel (ii), the axis of rotation is negative, (-n_1_). The dot product of the two axes of rotation vectors yields cos(θ), the measure of direction correlation (see Methods section for full details of the calculation).

In order to determine the local and mid-range influence of OmpF on the dynamics of different lipid populations, the correlation of the directions of lipid and protein displacements was calculated for lipid in the DS-DL-OmpF system, over different time jumps. The direction correlations were calculated separately for both (annular layer rich) DLPC lipids and for DSPC lipids (Fig 5). Significant protein and lipid displacement correlations were observed at both 0.2 ns and 2.0 ns time jumps, and for great circle distances beyond 15 nm. Both DLPC and DSPC showed high direction correlation with the movements of proteins at short distances (<0.5 nm) from the protein surface. In part, this is the result of long lifetime interactions of DLPC (in some case >150 ns) and DSPC (often <75 ns) lipids with the surface of the protein. At intermediate distances, between 0.5 and 1.5 nm from the surface of the protein, there existed a small amount of distortion in the correlations for DLPC lipids, which was absent in the correlations for DSPC lipids. This distortion appears at the approximate boundary of the DLPC and DSPC rich annular rings circumscribing OmpF (Figs 4 and 5Ai). Strikingly, at longer distances from the protein surface, from 1.5 nm and up to ~6 nm, there was still significant correlation between protein and lipid, though this was noticeably lower for the DLPC motion compared to DSPC. Longer timescale motions, up to 200 ns, although poorly sampled, also showed a similar, lower correlation for DLPC lipids than for DSPC lipids (Fig 5Aii). Interestingly, this longer-range region corresponded to the DSPC rich annular layer. Thus, OmpF influences the lipid dynamics and membrane composition up to 6 nm from its surface (~10.5 nm its COM), suggesting that dynamic protein-lipid complexes may be substantially larger than has previously been suggested [5,6].

### Protein aggregates induce long-range membrane undulations

**Fig 6:**
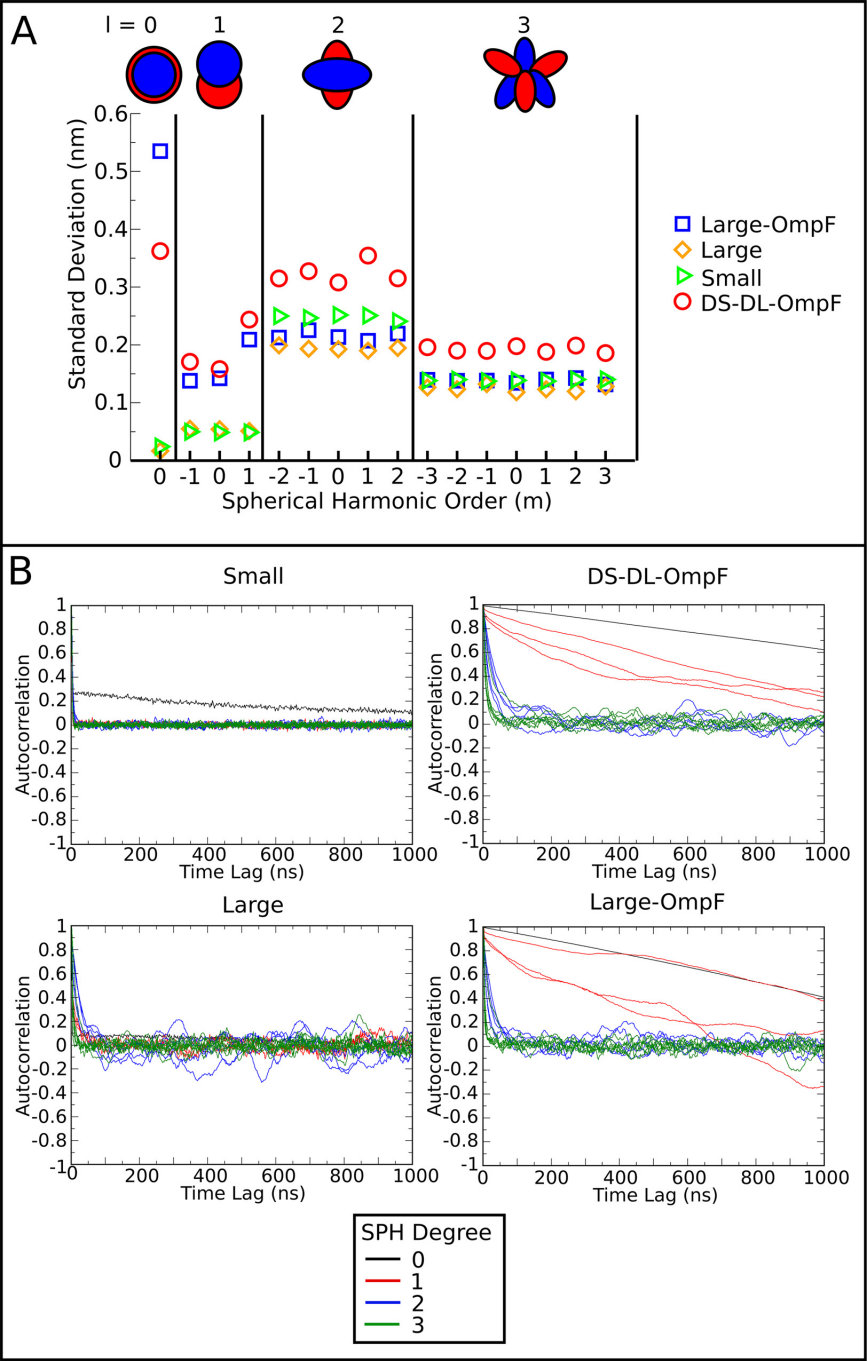
Spherical harmonics analysis to characterize global modes of undulation in each system. **(A)** The standard deviations of the fitted spherical harmonic across the trajectory, where *l* represents the degree and *m* the order. **(B)** The autocorrelation of the fitted spherical harmonics coefficients as a function of time for each vesicle, Small, Large, Large-OmpF and DS-DL-OmpF. The coefficients of each order (-l≤m≤l) of degree (l = 0,1,2,3) are shown in the same color but are presented as separate lines. The data presented in (A) and (B) has been obtained from frames spanning the whole trajectory.

The above analysis suggested that OmpF is associated with short- to mid-range changes in membrane structure, with an increased annular packing of lipids and tendency for lipid sorting, coupled to long-timescale, correlated motion between protein and lipid. However, this does not take into account global changes in vesicle morphology or long-range undulations. To quantify deformation of the vesicles, their shape was decomposed into a linear combination of real-valued spherical harmonics (*l* = 0 to *l* = 3 with −*l* ≤ *m* ≤ *l*, Eq 1). This enabled us to correlate global fluctuations with the size and composition of each vesicle system. Perhaps unsurprisingly, the largest shape descriptor was the first degree harmonic (*l* = 0, *m* = 0). Changes to this component represent fluctuations in the radius of the vesicle. The higher degrees of spherical harmonics exhibited orders of magnitude smaller coefficients than that of *l* = 0. As such, our analysis focused on the relative fluctuations in the coefficients. The SD of radial fluctuations were substantially larger in the OmpF vesicles, at 0.35 nm for Large-OmpF and 0.50 nm for DS-DL-OmpF, compared to Large and Small, SDs were both just ~0.02 nm (Fig 6A). There were also elevated fluctuations in the second degree harmonics (*l* = 1, *m* = −1,0,1) for the OmpF containing vesicles over the non-protein vesicles, with a SD of ~0.2 nm for Large-OmpF and DS-DL-OmpF versus ~0.05 for Large and Small. Fluctuations in these harmonics represent a movement of lipids from one hemisphere towards another in three orthogonal axes. It should be noted that the Large-OmpF and DS-DL-OmpF vesicles gradually deceased in radius by ~0.5 nm over the course of the simulations, as a result of the disruption to the lipid density of the vesicles after the initial insertion of the proteins. Consistent with this, the coefficients of the first and second degree harmonics exhibited long-timescale (> 1 μs) autocorrelation effects (Fig 6B), suggesting that this approach may represent a useful way of assessing convergence of such complex vesicle simulation systems.

The higher degree spherical harmonics, (*l* = 2, *m* = −2, −1,0,1,2) and (*l* = 3, *m* = −3, −2,−1,0,1,2), revealed elevated fluctuations in only the DS-DL-OmpF vesicle, with a SD of 0.3 nm compared to < 0.25 nm in Small, Large and Large-OmpF. These fluctuations represent flattening and elongation of the vesicle in several axes in degree *l* = 2, and more complex shape changes in degree *l* = 3. In contrast to the first two degree spherical harmonics, the coefficients in the higher degree spherical harmonics, *l* = 2 and *l* = 3, were not highly time-correlated, with autocorrelation times of < 100 ns, indicating that these enhanced dynamics are an inherent property of the bilayer conditions, and not associated with vesicle equilibration.

## Discussion

The current study aimed to investigate role of size, protein content, and lipid composition upon protein association, and the local and global dynamics of lipid vesicles. The interactions between integral membrane proteins and lipids are crucial for their respective spatial arrangement within cells. However, the molecular details of these interactions, and how they result in complex macroscopic arrangements, are still not fully understood. Several mechanisms including hydrophobic mismatch and curvature based sorting have been suggested as possible drivers of assembly and correlated motions. The results presented here suggest lipid compositions impact both the global and local dynamics of the membrane vesicle. On a local level, the OmpF trimers were able to alter the membrane properties by selecting out lipids that provide a “better interface” between the protein and the bulk lipids, in this case resulting in a thinning of the membrane close to the protein surface. The area of most bilayer thinning was found at “tips” of the trimer, which corresponds to the site of initial OmpF association during aggregation. Furthermore, this thinning was found to extend deeper into the bulk lipids at the interface between two OmpF trimers. Thus, the membrane alterations caused by proteins may provide a mechanism for long-range communication in protein sorting and aggregation. Indeed, the OmpF trimers were found to alter the dynamics of lipids up to 6 nm from their surface (~10.5 nm from the COM), a distance that is substantially further than previously detected [5, 6].

In addition, the global modes of undulation of the vesicles, assessed through spherical harmonics decomposition, were also shown to differ depending on the composition of the vesicles. Given the complexity of biological membranes, subtle changes in lipid composition may have implications for lipid and protein sorting under a curvature-based membrane-sorting model. It is possible, therefore, that changes to the fundamental shape components of the vesicles could modulate the patterns of lipid domain formation. High-frequency vibrations in any of these shape components would likely lead to the dispersal of membrane domains, while low frequency, or stable shapes, may promote demixing of lipids. Protein aggregation into domains in this context would consequently be affected. In the current study, the changes in the spherical harmonics had correlation times of up to 1 μs. These changes in shape are consistent with the aggregation times of the OmpF trimers. However, a causal relationship remains to be established. Protein induced shape changes of membranes may promote aggregation simply as a way to minimize curvature stress within the lipid bilayer. This coupled to lipid demixing, and the formation of a stable interface between the protein and lipid bilayer, may also contribute to aggregation, especially if it results in “channels” of bilayer deformation that can be remedied by a closer association of the proteins.

As a note on simulation methodology, we observed that the insertion of proteins during setup had long timescale implications for the fundamental dynamics of the vesicles. Like planar bilayer simulations, there appears to be scope for the development of tools that can reduce the equilibration time for vesicles after protein insertion. In addition, the decomposition of vesicles into their fundamental and orthogonal components represents an effective strategy for assessing convergence, and determining whether the vesicle membrane is well equilibrated after any disruption.

## Materials and Methods

### Vesicle construction

The vesicles were constructed using an in-house script that utilized the MDAnalysis python tools [31]. The center of mass of each lipid was placed on the surface of an ideal sphere, with the lipids spaced equally according to their average area per lipid in fluid phase, assuming a surface area of an ideal sphere located at the centre of mass (COM) of the lipid. The Small, Large and Large-OmpF systems contained a 3:1 mixture of palmitoleoyl-phosphatidyl-ethanolamine (POPE) and palmitoleoyl-phosphatidyl-glycerol (POPG) lipids, while the DS-DL-OmpF contained a 1:1 mix of di-stearoyl-phosphatidyl-choline (DSPC) and di-lauroyl-phosphatidyl-choline (DLPC). The proteins were inserted into the bilayer at equidistant positions from one another, and overlapping lipids were removed. The systems were energy minimized using the steepest decent algorithm for 1000 steps. The system was then solvated and neutralized with counter ions.

### Simulation parameters

All vesicle systems were simulated using the GROMACS package, version 4.0.7 [32–34]. The MARTINI force field version 1.4 was used for the lipid, water and ions [13], while the protein was modelled as described in [23–25] using an elastic network based potential in which all non-bonded backbone particles within ≤ 0.7 nm from one another were restrained to their initial distance via weak harmonic restraints, using a force constant of 1000 kJ mol^-1^ nm^-1^. During a 10 ns system equilibration performed, position restraints with a force constant of 1000 kJ mol^-1^ were applied to the backbone particles of the proteins in Large-OmpF and DS-DL-OmpF. All the vesicles were maintained at a constant temperature of 313 K using the Berendsen thermostat with a time constant of 10 ps [35]. A pressure of 1 bar was maintained using anisotropic pressure coupling with the Berendsen barostat with a time constant of 10 ps [35]. The van der Waals interactions were shifted to zero between 0.9 and 1.2 nm. Electrostatic interactions were truncated at 1.2 nm using a cut-off. The relative dielectric constant, *ε*, was set at 20. A timestep of 20 fs was used for integration. The neighbor list was updated every 10 steps during the simulations. A rhombic dodecahedron simulation cell, with a minimum distance of 2.5 nm between periodic images, was employed to reduce the number of solvent molecules in the system. The analysis of the simulations was performed using GROMACS tools and locally written scripts. The figures were generated using VMD [36] and R.

### General vesicle properties

The Mean Nearest Neighbor Distance (MNND) was measured as the minimum of each pairwise distance between lipid COMs. The average was taken over all lipid pairs in all frames to obtain the Mean Nearest Neighbor Distance. The frames were spaced at 2 ns intervals. The same procedure was used to calculate Mean Nearest Neighbor Great Circle Distance (MNNGC), but with the distance calculated as the great arc between each pair of lipid COMs.

### Identification of OmpF aggregation patterns

The interaction of two OmpF trimers was categorized into three categories termed tip-to-tip, tip-to-base and base-to-base. The interactions were assessed by measuring the inter subunit center of mass distances between each subunit of one trimer and the subunits on an opposing trimer. A subunit was deemed to have interacted with a subunit of another trimer if their center of mass distances were within 6.2 nm of one another. An interaction was categorized as tip-to-tip if a single subunit interacted with only one other subunit of another trimer. A tip-to-base interaction was recorded if two subunits of one trimer interacted with only one of another. A base-to-base interaction was recorded if there were three interactions between the two trimers.

### Membrane thickness

The protein’s great circle displacement and rotational motion about the vesicle surface normal was removed between trajectory frames. A grid was projected onto the surface of the vesicle, with the protein at the center of the grid. The lipids closest to each vertex were selected, and the distances of the headgroup particles from the vesicle COM were summed. The average was taken over all frames in the trajectory, with a 2 ns time interval between frames.

### Lipid distributions around OmpF

In a similar way to the membrane thickness calculations, a square grid was projected onto the vesicle surface following the removal of the protein’s great circle displacement and rotational motion. The occurrence of each lipid in each cell was counted and divided by the total number of observations.

### Spherical harmonic decomposition

In order to quantify the deformations of vesicles, their shape was decomposed into a linear combination of real-valued spherical harmonics (Eq 1). A linear combination of the first three degrees of spherical harmonics (*l* = 0 to *l* = 3 with −*l* ≤ *m* ≤ *l*) was fitted to the vesicle shape in each frame.

$$P_{fit}\left(r_{i},\theta ,\varphi \right)=c_{0}Y\left(l=0,m=0,\theta ,\varphi \right)+c_{1}Y\left(l=1,m=-1,\theta ,\varphi \right)+\cdots$$ (1)

Numerical minimization of the least squares distance (Eq 2) between the fitted shape and the positions observed in the respective frame of the trajectory yields a set of coefficients c_0_ to c_n_ as well as a residual distance that is not explained by this fit. We employed the scipy.optimize.minimize function with Powell’s method from SciPy version 0.14.1 [37] to fit 16 parameters for each individual spherical harmonic function up to *l* = 3 to each frame of the trajectories.

$$\text{min}\sum_{i}^{N}{\left(P_{i}^{fit}-P_{i}^{obs}\right)}^{2}$$ (2)

The observed coefficients quantify the contribution of the associated shape to the overall shape of the vesicle. The individual components were then analyzed with regard to their overall magnitude, their variance and the frequencies of their fluctuations. The residual distance was used as a quality metric for the fit. High residuals would indicate a shape that can be accurately described only by including higher order functions. Observed residuals were below 0.01 nm in all cases. In contrast to the procedures of Braun & Sachs [9] that employed an undulating reference surface and averaging on an equilateral grid, this approach takes each individual particle position into account.

### Direction correlations

The direction vector was considered to be the unitized displacement vector between a lipid’s position at *t*, and, *t* + Δ*t*. The correlation between two direction vectors was measured using the scalar product (Eq 3).

$$\text{cos}\;\theta =\frac{\vec{a}\cdot \vec{b}}{\left|a\right|\left|b\right|}$$ (3)

The angle between two unit vectors pointing in same direction is 0 rad, and thus, cos 0 = 1, while the angle between two vectors that point in opposite directions is *π* rad, and cos π = −1. Assuming the vesicle to be approximately spherical, the direction correlation of lipids in a vesicle are then calculated from their rotation axis vectors that describe great circles. Indeed, the angle between two great circles at the surface of a sphere, where they intersect, is equivalent to the angle between their axes of rotation. The rotation axis for a great circle between a molecules COM at *t*, and, *t* + Δ*t* is calculated using the vector product relation, where $\vec{a}$ and $\vec{b}$ are the two vectors representing the position of a particle at *t* and *t* + Δ*t*, *θ* is the angle of rotation, and $\vec{n}$ is the unit vector parallel to the axis of rotation (Eq 4).

$$\frac{\vec{a}\times \vec{b}}{\left|a\right|\left|b\right|\;\text{sin}\;\theta }=\vec{n}$$ (4)

The direction correlation between two molecules, *i* and *k*, is then the dot product between their two axes of rotation, represented by unit vectors ${\vec{n}}_{i}$ and ${\vec{n}}_{k}$ (Eq 5).

$$\text{cos}\;\theta ={\vec{n}}_{i}\cdot {\vec{n}}_{k}$$ (5)

Each value for the direction correlation was binned based on the great arc distance between the molecules. The average of the values in each bin was calculated.

## Supporting Information

S1 Fig Time series plots. (A) Vesicle radius, (B) lipid mean nearest neighbor distance (MNND), and (C) lipid mean nearest neighbor distance (great circle distance, MNNGC).
(DOCX)
