# Supplementary material for: Dynamics of Crowded Vesicles: Local and Global Responses to Membrane Composition
Source: PLoS One. 2016 Jun 16;11(6):e0156963. doi: 10.1371/journal.pone.0156963 (PMC4910979; doi:10.1371/journal.pone.0156963)
Supplement: S1 Fig — (A) Vesicle radius, (B) lipid mean nearest neighbor distance (MNND), and (C) lipid mean nearest neighbor distance (great circle distance, MNNGC). (DOCX) [file pone.0156963.s001.docx]

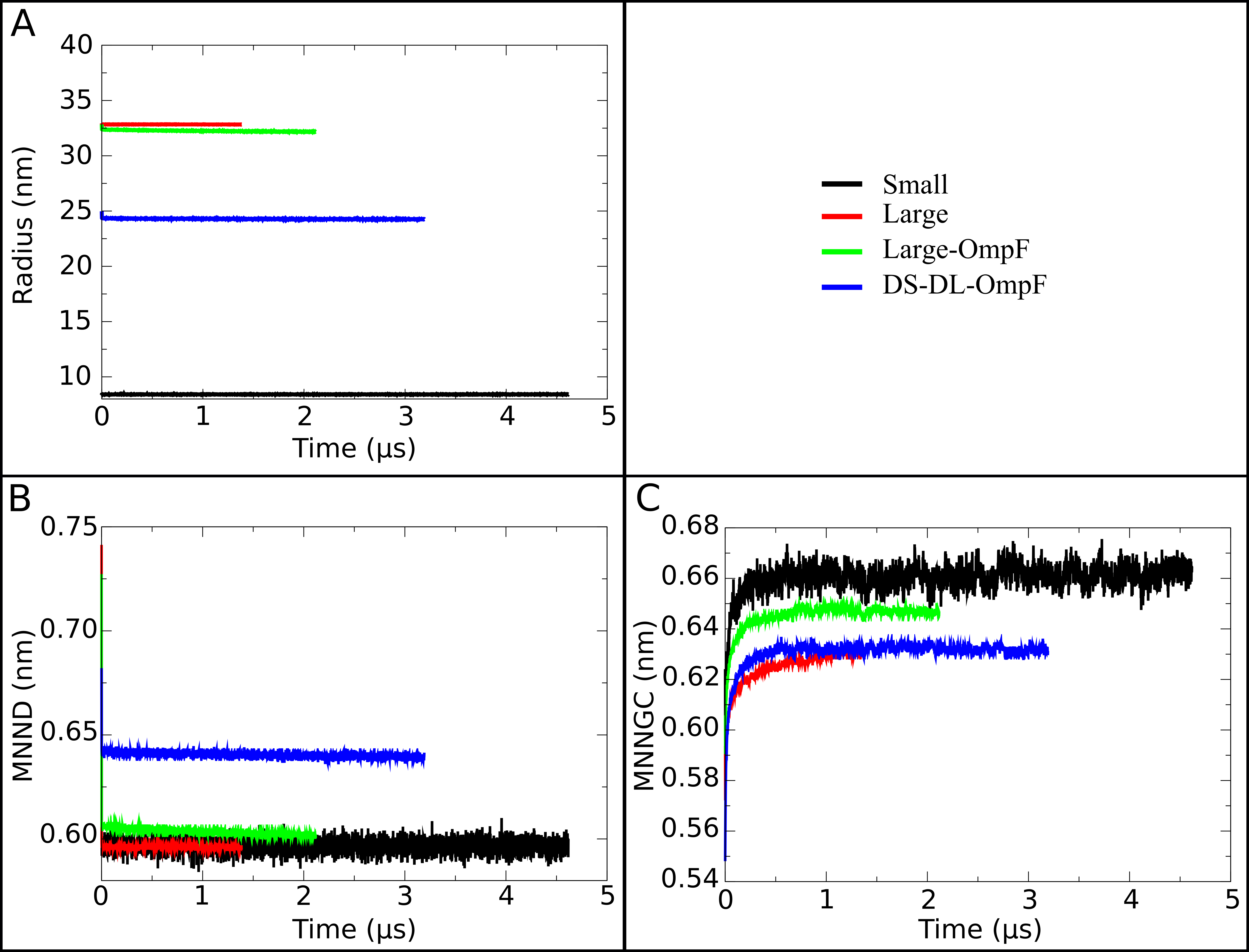


S1 Fig. Time series data for the (A) vesicle radius, (B) lipid mean nearest neighbor distance (MNND), and (C) lipid mean nearest neighbor distance (great circle distance, MNNGC).
